# Acute effects of lower-limb intermittent negative pressure on microcirculatory function in Tai Chi athletes: an exploratory study

**DOI:** 10.3389/fphys.2025.1668852

**Published:** 2026-01-13

**Authors:** Xiaoliang Wu, Lan Li, Qianqian Fan

**Affiliations:** Woosuk University, Wanju-gun, Republic of Korea

**Keywords:** intermittent negative pressure, microcirculation, recovery, Tai Chi, transcutaneousoxygen

## Abstract

**Objective:**

To determine whether a single 20-min bout of lower-limb intermittent negative pressure (INP) acutely improves microcirculation in competitive Tai Chi athletes.

**Methods:**

Twenty-eight male athletes (20.3 ± 1.0 years) underwent pre- and post-INP assessment of microvascular blood flow, moving blood-cell concentration, velocity, and transcutaneous oxygen (TcPO_2_) and carbon-dioxide (TcPCO_2_) tensions at the biceps brachii and vastus lateralis using laser-Doppler flowmetry and gas sensors. Vascular reserve was quantified after 5-min local heating. INP consisted of 30 s at −55 to −60 mbar followed by 10 s at atmospheric pressure for 20 min. Paired t-tests compared pre- and post-values; Cohen’s d gauged effect size.

**Results:**

At baseline, TcPO_2_ and TcPO_2_/TcPCO_2_ were lower in the lower limb (P < 0.001, d = 0.92–0.75). Post-INP, no significant changes occurred in the upper limb. In the lower limb, TcPO_2_ increased from 63.9 ± 10.2 to 77.4 ± 10.4 mmHg (P < 0.001, d = 1.31) and TcPO_2_/TcPCO_2_ rose from 1.8 ± 0.4 to 2.4 ± 0.7 (P < 0.001, d = 1.05); hemodynamic variables were unchanged.

**Conclusion:**

One session of lower-limb INP selectively enhances local oxygenation in Tai Chi athletes without affecting upper-limb microcirculation, offering a rapid, lower-limb-specific recovery strategy.

## 1 Introduction

Intermittent Negative Pressure (INP) is a non-invasive intervention method that promotes local blood circulation and microcirculatory function by periodically applying negative pressure (Bateman et al., 2016; Hoel et al., 2021a). Previous studies have shown that this technique can optimize local microcirculatory status by increasing vascular shear stress, improving endothelial cell function, and enhancing tissue oxygenation (Hoel et al., 2021b). In the field of sports medicine, INP has been applied to lower limb recovery, with mechanisms of action including improved blood flow perfusion, accelerated removal of metabolic waste, and enhanced tissue oxygen supply (Park et al., 2019). For example, studies have found that lower limb intermittent negative pressure significantly improves post-exercise hemodynamic parameters in athletes and shortens muscle fatigue recovery time (Hoel et al., 2021c). Additionally, this technology has been used to improve microcirculatory disorders in patients with peripheral arterial disease, indicating its broad potential in enhancing local vascular function (Soto et al., 2024). However, current research on the acute effects of INP on microcirculation in athletes of specific sports remains limited, particularly in sports requiring high lower limb stability and endurance.

Tai Chi, as a sport emphasizing slow, continuous movements with high lower limb load, places high demands on athletes' microcirculatory function (Li et al., 2022). Previous studies have indicated that microcirculatory function is closely related to athletic performance, with improved microcirculation optimizing muscle oxygen supply, accelerating the removal of metabolic waste, and delaying the onset of exercise-induced fatigue (Hoel et al., 2021a; Clément et al., 2024). For example, in endurance sports, enhanced microcirculatory function has been shown to improve exercise endurance and reduce the risk of muscle injury (Wiecha et al., 2021). However, Tai Chi athletes must maintain a low center of gravity posture for extended periods during competitions, with lower limb muscles continuously in an isometric contraction state, which can lead to localized blood flow restriction and oxygen deficiency, thereby affecting athletic performance and recovery efficiency (Li et al., 2023a; Li et al., 2023b). Although the importance of microcirculation in exercise science has been widely recognized, research on lower limb microcirculation in Tai Chi athletes remains unexplored. Therefore, exploring intervention methods that can rapidly improve lower limb microcirculation is of significant importance for enhancing the competitive performance and recovery efficiency of Tai Chi athletes.

The assessment of microcirculatory function typically involves hemodynamic parameters (such as blood flow velocity and blood cell concentration) as well as tissue oxygenation parameters (such as transcutaneous oxygen partial pressure and carbon dioxide partial pressure) (Wilk et al., 2020; Trotter et al., 2021). Previous studies have primarily focused on microcirculatory changes under static or single exercise conditions, with limited research specifically targeting Tai Chi, which requires complex lower limb coordination. This study utilized a laser Doppler blood flow meter and transcutaneous gas sensors to comprehensively assess microcirculatory changes in Tai Chi athletes before and after intermittent negative pressure intervention, with a focus on local oxygenation and vascular reserve capacity. This method not only comprehensively reflects the acute improvement effects on lower limb microcirculation but also provides targeted recovery strategies for Tai Chi athletes. Compared to traditional recovery methods (such as massage or cold therapy), intermittent negative pressure offers advantages such as non-invasiveness, strong targeting, and ease of operation, providing new research directions for the field of sports recovery.

This study aims to investigate the acute effects of a single session of intermittent negative pressure intervention on microcirculatory function in Tai Chi athletes, particularly changes in local oxygenation and hemodynamics. Unlike previous studies, this study is the first to apply intermittent negative pressure technology to Tai Chi athletes and uses a multi-indicator comprehensive evaluation of its effects. The results not only provide scientific evidence for lower limb recovery in Tai Chi athletes but also expand the application scope of intermittent negative pressure in sports medicine. Additionally, by comparing differences in microcirculatory responses between the upper and lower limbs, this study further validated the lower limb-specific effects of intermittent negative pressure, laying a theoretical foundation for its precise application in specialized sports. This innovative exploration not only fills a gap in microcirculatory research on Tai Chi Chuan but also provides new insights for optimizing athletes' recovery strategies. Based on the above background, we hypothesized that a single session of lower-limb intermittent negative pressure would acutely improve microcirculatory function in competitive Tai Chi athletes, with the effects being region-specific: (1) lower-limb oxygenation (as indicated by TcPO_2_ and TcPO_2_/TcPCO_2_ ratio) would significantly increase following INP intervention, whereas (2) upper-limb microcirculatory parameters would remain unchanged. This hypothesis aligns with the localized mechanical and physiological effects of INP and addresses the specific recovery needs of athletes engaged in sustained low-center-of-gravity postures.

## 2 Methods

### 2.1 Study design

A single-group pre–post design was employed to examine the acute effects of one 20-min bout of lower-body intermittent negative pressure on microcirculatory function in upper and lower limbs.

### 2.2 Participants

*A priori* sample size estimation was performed using G*Power software (version 3.1.9.7) based on data from a pilot study involving 8 Tai Chi athletes (not included in the final sample) who underwent the same INP protocol. The primary outcome was the change in transcutaneous oxygen tension (TcPO_2_) at the vastus lateralis. The pilot study yielded a mean pre-post difference of 10.5 mmHg with a standard deviation of 8.2 mmHg, corresponding to an effect size (Cohen’s *d*) of 1.28.

For a paired-sample *t*-test with an alpha level of 0.05 (two-tailed) and a statistical power of 0.95, a minimum sample size of n = 12 was required to detect a similar effect. To account for potential attrition, technical failures, or data exclusion due to movement artifacts, and to ensure robust subgroup comparisons (e.g., by athletic level), we increased the sample size to n = 28. This sample size also aligns with recommendations for pilot or exploratory studies in sports physiology where effect sizes are typically large (*d* > 0.8) and within-subject designs are employed (Table 1).

**TABLE 1:** Baseline comparison between upper (biceps brachii) and lower (quadriceps femoris) limbs (n = 28).

Indicator	Site	Baseline (M±SD)	t-value	P-value	Cohen’s d
MBP (PU)	Biceps brachii	12 ± 4	0.84	0.407	0.16
	Quadriceps femoris	11 ± 4			
CMBC (AU)	Biceps brachii	117 ± 69	1.08	0.289	0.21
	Quadriceps femoris	100 ± 54			
AVBC (mm/s)	Biceps brachii	13 ± 6	0.00	1.000	0.00
	Quadriceps femoris	13 ± 5			
TcPO_2_ (mmHg)	Biceps brachii	72.8 ± 9.4	5.07	<0.001*	0.92
	Quadriceps femoris	63.9 ± 10.2			
TcPCO_2_ (mmHg)	Biceps brachii	35.0 ± 2.7	2.73	0.011	0.52
	Quadriceps femoris	36.9 ± 2.6			
TcPO_2_/TcPCO_2_	Biceps brachii	2.1 ± 0.4	4.38	<0.001*	0.75
	Quadriceps femoris	1.8 ± 0.4			

MBP, microcirculatory blood perfusion; CMBC, circulating blood cell concentration; AVBC, average blood cell velocity; PU, perfusion unit; AU, arbitrary unit. P-values were FDR-corrected, indicating statistical significance after correction (P < 0.05). *

### 2.3 Experimental protocol

The trial was conducted on a complete rest day during the summer training camp. Participants arrived 1 h post-prandial, donned standardized clothing, and rested supine for 15 min to acclimatize to the laboratory environment (25.0 °C ± 0.5 °C, 50% ± 5% relative humidity).

1. Pre-intervention assessment. After anatomical landmarks were identified on the right biceps brachii and right vastus lateralis, a laser-Doppler flowmeter (PeriFlux System 6000, Perimed, Sweden) with integrated oxygen and carbon-dioxide probes recorded 3 min baseline values for microcirculatory blood perfusion, concentration of moving blood cells, average blood-cell velocity, transcutaneous oxygen tension and transcutaneous carbon-dioxide tension. A built-in heater (44 °C) was then activated for 5 min; the difference between the final 2 min of heating and the preceding baseline defined vascular reserve.
2. Lower-body intermittent negative pressure. Participants wore a neoprene waist seal and lay supine inside a VACUSPORT chamber (Vacumed, Germany) with the body below the iliac crest enclosed. The protocol comprised 30 s of negative pressure (−55 to −60 mbar) followed by 10 s of atmospheric pressure, repeated for 20 min. Volunteers were instructed to remain awake and relaxed without voluntary leg contraction. The 20-min duration of the intermittent negative pressure (INP) protocol was selected based on previous studies demonstrating acute microcirculatory and hemodynamic adaptations within this time frame. Investigations using lower-limb negative pressure in both clinical and athletic populations have reported significant improvements in tissue oxygenation and vascular function after 15–30 min of intervention, with 20 min representing a common and well-tolerated exposure that balances efficacy with participant comfort and practicality in a sports-setting (Bonanno, 2022).
3. Post-intervention reassessment. Within 60 s of intervention cessation, identical measurements were repeated at the same sites with probe repositioning error <2 mm. All assessments were performed by the same investigator.

### 2.4 Control measures

To ensure the internal validity and reproducibility of the experiment, rigorous control measures were implemented across three domains.

#### 2.4.1 Subject management and standardization

Diet and Activity Control: Participants were instructed to avoid caffeine, alcohol, and heavy meals for at least 12 h prior to testing. All tests were conducted after a 1-h postprandial period to standardize metabolic state. Strenuous physical activity was prohibited for 48 h prior to the experiment.

Posture and Movement Restriction: Throughout the pre- and post-intervention assessment periods, participants maintained a supine position with limbs relaxed and supported. A tri-axial accelerometer (integrated into the PeriFlux system) was used to monitor and exclude any data segments coinciding with limb movement (>0.05 g), ensuring measurements were taken under true resting conditions.

Physiological State Monitoring: Skin temperature at the measurement sites was continuously monitored. Any trial with a skin-temperature drift exceeding 0.5 °C during the baseline or intervention period was discarded and repeated after re-stabilization.

#### 2.4.2 Intervention protocol administration

Equipment Calibration and Standardization: The VACUSPORT negative-pressure chamber was calibrated daily according to the manufacturer’s specifications. The negative-pressure cycle (30 s at −55 to −60 mbar/10 s at atmospheric pressure) was controlled by the built-in automated system to ensure precise timing and pressure application across all participants.

Participant Positioning and Compliance: The neoprene waist seal was checked for airtightness for each participant. During the 20-min INP session, participants were instructed to remain awake, relaxed, and to avoid any voluntary muscle contractions in the lower limbs. The investigator verbally confirmed compliance throughout the session.

Probe Placement and Reproducibility: Anatomical landmarks (midpoint of the muscle belly for both biceps brachii and vastus lateralis) were marked with a dermatological pen to ensure identical probe placement for pre- and post-intervention measurements. Probe-skin contact pressure (∼2 N cm^−2^) and orientation (perpendicular to the skin surface) were strictly maintained using a calibrated force gauge and a goniometer, respectively. Repositioning error between measurements was kept below 2 mm.

#### 2.4.3 Environmental control

Laboratory Conditions: The experiment was conducted in a dedicated climate-controlled laboratory maintained at 25.0 °C ± 0.5 °C and 50% ± 5% relative humidity. These conditions were verified using a calibrated digital thermo-hygrometer at the beginning of each testing day.

Acclimatization and Rest: Upon arrival, participants donned standardized lightweight clothing (shorts and t-shirt) and rested in a supine position for a standardized 15-min period to achieve cardiovascular and thermal equilibrium before any baseline measurements were initiated.

Minimization of External Stimuli: The testing room was kept quiet and free from unnecessary personnel. Lighting was kept constant and dim to minimize auditory and visual distractions that could influence autonomic nervous system activity and, consequently, microcirculatory parameters.

### 2.5 Measurements

Microcirculatory variables were acquired by laser-Doppler flowmetry combined with transcutaneous gas sensors. Probe–skin contact pressure (∼2 N cm^−2^) and orientation (90° perpendicular) were maintained across trials.

Microcirculatory blood perfusion was expressed in perfusion units (1 PU = 10 mV kHz^−1^). Signals sampled at 32 Hz were band-pass filtered (0.02–12 Hz) and analysed using PF4 software. Baseline and heating values were averaged from 3 min to 2 min epochs; the difference defined perfusion reserve. Segments with coefficient of variation >15% were discarded.

Concentration of moving blood cells was derived from the integrated Doppler power spectrum; values < 20 arbitrary units were excluded. Average blood-cell velocity was computed from the weighted mean Doppler frequency shift; velocity histograms were inspected for artefacts.

Transcutaneous oxygen tension and transcutaneous carbon-dioxide tension were measured with integrated Clark-type and Severinghaus-type electrodes heated to 44 °C. After 5-min equilibration and two-point calibration, 3 min baseline and 2 min heating values were averaged; differences defined respective reserves. The TcPO_2_/TcPCO_2_ ratio was calculated as an index of local ventilation–perfusion matching. TcPO_2_ < 40 mmHg prompted probe re-fixation.

Quality assurance. Skin temperature was monitored continuously; trials with drift >0.5 °C were repeated. A tri-axial accelerometer excluded data coinciding with limb movement >0.05 g. Final datasets required ≥90% valid data. All data were exported as.csv files via Perisoft 3.2.

### 2.6 Statistical analysis

Statistical analysis was conducted using SPSS software (version 26.0; IBM Corp., Armonk, NY, United States). Normality of all outcome variables—including microvascular blood perfusion (MBP), concentration of moving blood cells (CMBC), average blood-cell velocity (AVBC), transcutaneous oxygen tension (TcPO_2_), transcutaneous carbon-dioxide tension (TcPCO_2_), and their derived ratios (TcPO_2_/TcPCO_2_) and vascular reserves (ΔMBP)—was assessed using Shapiro–Wilk tests, with all variables satisfying normality assumptions (*p* > 0.05).

Between-limb comparisons at baseline were performed using independent-samples *t*-tests. Within-limb pre-post intervention changes were evaluated with paired-samples *t*-tests. To account for multiple comparisons across all measured microcirculatory parameters (8 primary variables per limb), a false discovery rate (FDR) correction using the Benjamini–Hochberg procedure was applied, with an overall significance threshold set at α = 0.05 (two-tailed). Post-hoc interpretation of individual comparisons is based on FDR-adjusted *p*-values.

Effect sizes were quantified using Cohen’s *d*, with 95% confidence intervals (CI) calculated via bootstrapping (1000 resamples). Effect magnitudes were interpreted as small (0.2–0.49), medium (0.5–0.79), or large (≥0.8). Sample size was estimated *a priori* using G*Power 3.1, based on pilot data (ΔTcPO_2_ ∼ 10 mmHg, SD ∼ 8 mmHg, *d* ∼ 1.25). For a paired *t*-test with α = 0.05 and power (1-β) = 0.95, a minimum of n = 12 was required. To accommodate data variability, movement artefacts, and subgroup stability, 28 participants were recruited. All data are presented as mean ± standard deviation (SD).

## 3 Results

### 3.1 Baseline microcirculatory characteristics between regions

At rest, transcutaneous oxygen tension was significantly lower in the vastus lateralis than in the biceps brachii (63.9 ± 10.2 mmHg versus 72.8 ± 9.4 mmHg, t = 5.07, P < 0.001, d = 0.92), and the corresponding TcPO_2_/TcPCO_2_ ratio followed the same pattern (1.8 ± 0.4 versus 2.1 ± 0.4, t = 4.38, P < 0.001, d = 0.75). Microcirculatory blood perfusion, concentration of moving blood cells and average blood-cell velocity did not differ between the two sites (all P > 0.05). Thus, even under quiet resting conditions the lower limb already exhibited a markedly inferior local oxygenation status compared with the upper limb.

### 3.2 Acute microcirculatory response in the biceps brachii

Following the 20-min intermittent lower-body negative-pressure session, no variable measured at the biceps brachii changed significantly. Baseline microcirculatory blood perfusion declined from 12 ± 4 PU to 8 ± 2 PU (t = 2.56, P = 0.017), yet the associated effect size remained small (d = 0.49). The perfusion reserve (ΔMBP) was also unchanged (107 ± 44 PU pre-intervention versus 101 ± 38 PU post-intervention, t = 0.71, P = 0.484). Likewise, concentration of moving blood cells, average blood-cell velocity, TcPO_2_, TcPCO_2_ and the TcPO_2_/TcPCO_2_ ratio were all statistically indistinguishable from their pre-intervention values (all P > 0.05), with effect sizes uniformly below 0.5. Collectively, these findings indicate that a single bout of lower-body intermittent negative pressure exerts, at best, a negligible acute influence on upper-limb microcirculation (Table 2).

**TABLE 2:** Changes in biceps brachii microcirculation before and after intervention (n = 28).

Indicator	Time point	Baseline (M±SD)	Post-heating (M±SD)	Vascular reserve Δ (M±SD)	t-value	P-value	Cohen’s d
MBP (PU)	Pre-intervention	12 ± 4	119 ± 49	107 ± 44	2.56	0.017	0.49
	Post-intervention	8 ± 2	109 ± 38	101 ± 38			
CMBC (AU)	Pre-intervention	117 ± 69	301 ± 55	184 ± 59	0.37	0.712	0.07
	Post-intervention	93 ± 20	273 ± 59	180 ± 59			
AVBC (mm/s)	Pre-intervention	13 ± 6	40 ± 10	27 ± 14	1.01	0.322	0.19
	Post-intervention	11 ± 4	41 ± 11	30 ± 13			
TcPO_2_ (mmHg)	Pre-intervention	72.8 ± 9.4	—	—	1.57	0.128	0.22
	Post-intervention	71.0 ± 6.6	—	—			
TcPCO_2_ (mmHg)	Pre-intervention	35.0 ± 2.7	—	—	1.38	0.179	0.20
	Post-intervention	36.2 ± 3.6	—	—			
TcPO_2_/TcPCO_2_	Pre-intervention	2.1 ± 0.4	—	—	1.07	0.296	0.25
	Post-intervention	2.0 ± 0.3	—	—			

### 3.3 Acute microcirculatory response in the vastus lateralis

In contrast, the vastus lateralis demonstrated pronounced functional improvement after the intervention. Transcutaneous oxygen tension rose from 63.9 ± 10.2 mmHg to 77.4 ± 10.4 mmHg (t = 8.08, P < 0.001, d = 1.31), and the TcPO_2_/TcPCO_2_ ratio increased from 1.8 ± 0.4 to 2.4 ± 0.7 (t = 4.73, P < 0.001, d = 1.05). Although TcPCO_2_ showed a numerical reduction from 36.9 ± 2.6 mmHg to 34.0 ± 5.3 mmHg, this difference did not survive Bonferroni correction (t = 2.26, P = 0.032). None of the haemodynamic variables—microcirculatory blood perfusion, concentration of moving blood cells or average blood-cell velocity—changed significantly (all P > 0.05). Taken together, these results demonstrate that a single session of intermittent lower-body negative pressure acutely enhances lower-limb microcirculatory function primarily through improved local oxygenation (Table 3).

**TABLE 3:** Changes in quadriceps femoris microcirculation before and after intervention (n = 28).

Indicator	Time point	Baseline (M±SD)	Post-heating (M±SD)	Reserve Δ (M±SD)	t-value	P-value	Cohen’s d
MBP (PU)	Pre-intervention	11 ± 4	120 ± 56	109 ± 56	0.78	0.440	0.15
	Post-intervention	12 ± 5	126 ± 40	113 ± 39			
CMBC (AU)	Pre-intervention	100 ± 54	233 ± 65	133 ± 56	3.10	0.005*	0.59
	Post-intervention	106 ± 38	270 ± 39	163 ± 42			
AVBC (mm/s)	Pre-intervention	13 ± 5	52 ± 14	40 ± 15	1.98	0.058	0.38
	Post-intervention	12 ± 4	48 ± 14	36 ± 15			
TcPO_2_ (mmHg)	Pre-intervention	63.9 ± 10.2	—	—	8.08	<0.001*	1.31
	Post-intervention	77.4 ± 10.4	—	—			
TcPCO_2_ (mmHg)	Pre-intervention	36.9 ± 2.6	—	—	2.26	0.032	0.33
	Post-intervention	34.0 ± 5.3	—	—			
TcPO_2_/TcPCO_2_	Pre-intervention	1.8 ± 0.4	—	—	4.73	<0.001*	1.05
	Post-intervention	2.4 ± 0.7	—	—			

## 4 Discussion

### 4.1 Lower-limb hypoxia at rest in Tai Chi athletes

The results of this study indicate that, at rest, the local oxygenation status of the lower limbs (vastus lateralis) in Tai Chi athletes is significantly lower than that of the upper limbs (biceps brachii), as evidenced by significantly reduced TcPO_2_ and TcPO_2_/TcPCO_2_ ratios, while microcirculatory hemodynamic parameters (MBP, CMBC, AVBC) show no significant differences. This finding is consistent with previous studies on lower limb microcirculatory characteristics. Previous studies have shown that due to gravitational effects and the metabolic characteristics of lower limb muscles, the partial pressure of oxygen in lower limb tissues is typically lower than that in upper limbs during rest, particularly in athletes who engage in prolonged lower limb load-bearing exercises (Bonanno, 2022). The TcPO_2_/TcPCO_2_ ratio results from this study further support this view, suggesting that lower limbs may have higher metabolic demands or relatively lower perfusion efficiency (McDermott et al., 2021). Additionally, the lack of significant differences in hemodynamic indicators between groups may be related to compensatory mechanisms in whole-body blood flow distribution during rest, consistent with reports of microcirculatory adaptive changes in athletes (Menêses et al., 2024). The results of this study not only validate regional differences in lower limb oxygenation status among Tai Chi athletes but also provide theoretical support for the necessity of lower limb-specific interventions (such as INP) in future research. Future studies could further explore whether these differences are related to sport-specific characteristics (such as the low center of gravity posture in Tai Chi) and provide new directions for optimizing lower limb recovery strategies for athletes.

### 4.2 Acute effects of INP on lower-limb microcirculation: oxygenation improvement without hemodynamic change

The results of this study indicate that a single session of intermittent negative pressure (INP) intervention significantly improves local oxygenation function in the lower limbs (vastus lateralis) of Tai Chi athletes, as evidenced by significant increases in TcPO_2_ and the TcPO_2_/TcPCO_2_ ratio, with effect sizes (Cohen’s d) exceeding 1.0, demonstrating a strong intervention effect. Notably, this improvement was primarily reflected in tissue oxygenation indices, while traditional hemodynamic parameters (MBP, CMBC, AVBC) did not undergo significant changes. This finding is consistent with some previous studies where negative pressure intervention improved oxygenation parameters without altering conventional hemodynamic measures (Anderson et al., 2021; Iacovelli et al., 2024). The dissociation between oxygenation improvement and unchanged hemodynamics suggests that INP may enhance local oxygen availability through mechanisms other than increased bulk blood flow. However, the specific physiological pathways underlying this dissociation—whether related to improved oxygen diffusion, microvascular redistribution, or other factors—remain to be elucidated through studies incorporating direct mechanistic assessments.

### 4.3 Regional specificity of INP: no acute effect on upper-limb microcirculation

Additionally, the results of this study indicate that a single 20-min lower limb intermittent negative pressure (INP) intervention did not significantly affect microcirculatory function in the upper limbs (biceps brachii) of Tai Chi athletes, with no statistically significant differences in all measured parameters (MBP, CMBC, AVBC, TcPO_2_, TcPCO_2_, and TcPO_2_/TcPCO_2_), and the effect sizes (Cohen’s d) were all below 0.5. This finding aligns with previous observations that negative pressure interventions exert primarily local or segmental effects (Fejfarová et al., 2021). Although MBP showed a slight decrease (d = 0.49) in this study, combined with the absence of significant changes in vascular reserve (ΔMBP) and other indicators, this further supports the localized nature of INP’s effects. This regional specificity may be related to the mechanical scope of negative pressure application and the segmental nature of neurovascular regulation (Betik et al., 2021). Future studies may further explore the potential effects of different negative pressure parameters on microcirculation in distant sites to optimize the design of intervention protocols.

### 4.4 Practical implications and future research directions

This study provides important evidence for developing recovery strategies tailored to specific sports. The selective improvement in lower-limb oxygenation, coupled with the absence of upper-limb effects, underscores the targeted nature of INP as a potential recovery modality for activities with high lower-limb demands (Broatch et al., 2021). These preliminary findings suggest that INP could offer a non-invasive approach to acutely enhance local tissue oxygenation in athletes. However, given the exploratory design and lack of mechanistic measurements, these observations require confirmation in controlled trials. Future research should investigate the physiological mechanisms underlying the oxygenation-hemodynamics dissociation, possibly by incorporating assessments of microvascular function, oxygen extraction, or endothelial biomarkers. Additionally, studies examining the effects of different INP parameters, multiple sessions, and functional performance outcomes are needed to establish optimal protocols for sports applications.

### 4.5 Study limitations

This study has several limitations. First, the single-group pre-post design lacks a control or sham intervention, which prevents definitive causal attribution of the observed changes to INP. Time-related effects, measurement habituation, or natural variability cannot be ruled out. Therefore, the findings should be interpreted as exploratory and hypothesis-generating. Second, only male Tai Chi athletes were studied, limiting generalizability. Third, outcomes were restricted to immediate microcirculatory measures without functional performance data. Future randomized controlled trials with sham comparators, mixed-sex samples, and integrated performance metrics are needed to confirm and extend these preliminary observations.

## 5 Conclusion

This exploratory study observed that a single session of lower-limb intermittent negative pressure (INP) was associated with acute increases in local oxygenation parameters at the vastus lateralis in competitive Tai Chi athletes. Notably, corresponding changes were not detected at the biceps brachii. While this suggests a localized pattern of response, the absence of a control or sham intervention precludes a definitive conclusion regarding regional specificity. The observed dissociation between improved oxygenation and unchanged hemodynamics indicates that the acute effects of INP in this cohort may involve mechanisms distinct from simple increases in bulk blood flow.

The findings offer preliminary support for further investigation of INP as a potential non-invasive modality for enhancing lower-limb tissue oxygen availability in athletes engaged in sustained lower-limb effort. The results may also inform future research into its applicability for the general public—such as individuals participating in hiking, cycling, or occupations involving prolonged standing—where localized lower-limb recovery is desirable. However, randomized controlled trials incorporating sham conditions are required to confirm causality, establish regional specificity, and determine optimal application parameters across different populations and activity contexts. Future studies should also examine long-term adaptations, functional performance outcomes, and the underlying physiological mechanisms of INP.

## Data Availability

The original contributions presented in the study are included in the article/supplementary material, further inquiries can be directed to the corresponding author.
